# Photoprotective effect of *Astragalus membranaceus* polysaccharide on UVA-induced damage in HaCaT cells

**DOI:** 10.1371/journal.pone.0235515

**Published:** 2020-07-21

**Authors:** Qiong Li, Depeng Wang, Donghui Bai, Chao Cai, Jia Li, Chengxiu Yan, Shuai Zhang, Zhijun Wu, Jiejie Hao, Guangli Yu

**Affiliations:** 1 Key Laboratory of Marine Drugs, Ministry of Education, School of Medicine and Pharmacy, Ocean University of China, Qingdao, China; 2 Shandong Provincial Key Laboratory of Glycoscience and Glycotechnology, School of Medicine and Pharmacy, Ocean University of China, Qingdao, China; 3 Laboratory for Marine Drugs and Bioproducts, Pilot National Laboratory for Marine Science and Technology (Qingdao), Qingdao, China; 4 Infinitus (China) Company Ltd., Guangzhou, China; Annamalai University, INDIA

## Abstract

**Background:**

The skin provides a predominant barrier against chemical, physical and microbial incursion. The intemperate exposure to ultraviolet A (UVA) radiation can cause excessive cellular oxidative stress, leading to skin damage, proteins damage and mitochondrial dysfunction. There is sufficient evidences supporting the proposal that mitochondria is highly implicated in skin photo-damage.

**Methods:**

In the present study, a polysaccharide isolated from *Astragalus membranaceus* was further purified to be an α-glucan, which was further investigated its beneficial influence on UVA-induced photo-damage in HaCaT cells.

**Results:**

Our results showed that the purified *Astragalus membranaceus* polysaccharide (AP) can protect HaCaT cells from UVA-induced photo-damage through reducing UVA-induced intracellular ROS production and mitochondrial membrane potential, thereby altering ATP content. It was found that the UVA induced damage in HaCaT cells could be effectively restored by co-treatment with AP.

**Conclusions:**

AP exhibited promising potential for advanced application as multifunctional skin care products and drugs.

## Introduction

As well all know that UV radiation is the main factor that accelerates skin photo-damage and accelerates the natural aging process. It has been fully reported that 90% of skin damage is caused by UV radiation [1–3]. The UV scope consists of three wavelength ranges: UVC (200–280 nm), UVB (280–320 nm) and UVA (320–400 nm) [4]. 90% of the solar radiation reaching the Earth's surface is composed of UVA, and it penetrates the skin deeper than UVB [5, 6]. And long-term radiation to UVA radiation can cause numerous skin lesions, such as photosensitivity skin diseases and cancer [7].

The connection of aging phenotypes to cellular senescence and dysfunction has been reported in many researches [8–10]. Mitochondria can produce ATP which is used as chemical energy for most eukaryotic cells, and they also control cell functions related to differentiation, cell signaling, cell growth and death [11].Mitochondria are the main source of ROS production though OXPHOS activity in the inner mitochondrial membrane (mainly at complex I and II), which in turn is the major target of ROS damage [11, 12].

Mitochondrial DNA (mtDNA) attaches to the matrix of the inner membrane of the mitochondria, which is extremely sensitive to ROS in the mitochondria. Meanwhile, it was also reported that our body’s reactive oxygen species (ROS), mitochondrial dysfunction and mitochondrial DNA (mtDNA) damage increased with photo-damage [13, 14]. UVA radiation could induce mitochondrial dysfunction which directly contributes to photo-damage, and lead to the generation of mutations in mitochondrial [15, 16]. ROS still act on cardiolipin (CL), which can be oxidized and then converted into lipid second-messengers to advocate oxidative stress and disrupt mitochondrial metabolism [17, 18]. Mitochondrial dysfunction can induce cellular calcium influx and decreases ATP production, which could influence the cell membrane permeability in a feedback loop and mitochondrial membrane potential [19]. Indeed, there is increasing interest in protecting mitochondria from UVA induced ROS damage for the precaution and treatment of UVA-induced photo-damage. Consequently, a number of recent researches discovered that the plant extracts and natural products are particularly effective in the protection of skin from photo-damage by targeting to mitochondria, such as proanthocyanidins, carotenoids, lycopene [20–23]. Some reports have reported that glucan how potent inhibitory effects against UV-induced skin photo-ageing, which was induced by UVB irradiation through ROS. Such as alpha1-4-glucan, β-1,3/1,6-glucan and β-(1,3)-β-(1,4)-glucan [24–26].

*Astragalus membranaceus* is a traditional Chinese herbal medicine. Many studies have shown that *Astragalus membranaceus* exhibits multiple physiological functions that confer anti-oxidation, anti-inflammatory, anti-diabetic and anti-tumor. Among them, Astragalus flavonoids, saponin and polysaccharides are investigated to be the main active ingredients that exert those diverse functions [27, 28]. Furthermore, polysaccharides, as one of the major components in *Astragalus*, have also been widely investigated for their significant bioactivities. However, there is no systematic research previously reported the anti-photo-damage effect of *Astragalus* polysaccharide. Skin health promotion effects of natural glucan derived from cereals and microorganisms have been studied before, but their functional mechanism have rarely reported especially through mitochondrial mediation.

In our study, a representative polysaccharide was isolated and purified from *Astragalus membranaceus*. The total sugar, protein content and molecular weight (*M*_w_) of AP were characterized to be 64.9%, 20.1% and 32 kDa, respectively. The structural character of major component of AP was identified to be α-(1→4)-glucan by infrared, 1D and 2D-NMR spectra. The protective effects of AP on UVA-induced photo-damage HaCaT cells was evaluated.

## Materials and methods

### Materials and reagents

*Astragalus membranaceus* (from Gansu Province) was provided by Infinitus (China) Company Ltd. (Guangzhou, China). MEM medium, fetal bovine serum (FBS) were purchased from Hyclone (Logan, UT), penicillin Invitrogen (Carlsbad, CA) and streptomycin were purchased from Gibco (Grand Island, NY).

### Extraction and purification of AP

The dried roots of *Astragalus membranaceus* were pulverized and sieved through 60-mesh to obtain fine powder. Deionizer water was added to *Astragalus* powder at the ratio of 20/1 (v/m), extracted by reflux at 80°C. The mixture was centrifuged at 0°C, and then the supernatant was collected. Then, deionized water was added to the residue at the ratio of 15/1 (v/m). The mixture was extracted repeatedly by reflux at 80°C for 3h and centrifuged again. The supernatant was combined and concentrated by using a vacuum rotary evaporator. Crude polysaccharide was obtained after precipitation with four volumes of absolute ethanol and freeze-dried.

The crude polysaccharide was purified on the Q-Sepharose Fast Flow (QFF) column with gradient elution of 0–2 M NaCl solutions at a flow rate of 3.0 ml/min. The purified polysaccharide was collected at 0 M NaCl, which was named as *Astragalus membranaceus* polysaccharide (AP).

### Total sugar and protein contents of AP

The total sugar content in AP was measured by using the standard phenol-sulfuric acid method [29]. The protein content in AP was determined by using the Coomassie Brilliant Blue method [30].

### Molecular weights and monosaccharide composition analysis of AP

The weight-averaged molecular weight (M_w_) of AP were determined by high performance gel permeation chromatography combined with multi-angle laser light scattering (HPGPC-MALLS) on an Agilent 1260 chromatographic instrument. The AP was dissolved in 0.1 mol/L Na_2_SO_4_ solution at a concentration of 5 mg/ml. Next, the AP(100ul) was added to a Shodex OHpak column(35°C) and washed with 0.1 mol/L Na2SO4 solution at 0.6 ml/min. And then, the signal was detected by G1362A refractive index detector (RID) and MALLS (Dawn Heleos-II, Wyatt technologies, USA). The M_w_ of AP was calculated by Astra 5.3.4.20 software.

Monosaccharide composition of AP was determined by a pre-column derivatives 1-phenyl-3-methyl-5-pyrazolone(PMP)-high performance liquid chromatography (HPLC) method. The polysaccharide was hydrolyzed by TFA. Then, the sample was heated with PMP at 70°C for 1h. The sample was analyzed by using a C_18_ column at 1 ml/min, which was determined at 245 nm.

### Fourier Transform in Infrared Spectroscopy (FT-IR) and Nuclear Magnetic Resonance (NMR) analysis of AP

For FT-IR analysis,100 mg dried KBr was mixed with the dried AP (3–4 mg) and then monitored by a Nexus 470 FT-IR spectrometer (Thermo Electron) at 400–4000 cm−1 under dry air. For 1H-NMR and ^13^C-NMR analyses, the AP was dissolved in D2O, and t freeze-dried twice. Spectra were analyzed by using JNM-ECP 600 MHz equipment (JNM-ECP 600, Jeol, Japan) at 25°C. The data was analyzed by using the MestReNova software.

### Cell culture and treatments

HaCaT cells, from American Type Culture collection (ATCC), were grown in a humidified (5% CO2, 95% air) atmosphere with MEM medium containing 10% fetal bovine serum, and protected by 2 mM L-glutamine, 100 U/ml penicillin/streptomycin. HaCaT cells seeded in 96-well micro plate were cultured at 37°C for 12h. Then, the HaCaT cells grown in MEM supplemented with various concentrations of AP at 37ºC and 5% CO_2_ for 48 hours. The medium was discarded, serum-containing MEM was added and exposed in 30 J/cm^2^ ultraviolet irradiation.

### MTT assay

After the cells were treated for 24h, the HaCaT cell viability was analyzed by using MTT assay. Then, 20μl/well of MTT solution (5 mg/ml in PBS buffer) was added for 4h. The medium was aspirated, and then replaced with DMSO for dissolving the Potassium salt. The color intensity of the Potassium solution, which reflects the cell growth condition, was measured at 570 nm using a micro plate spectrophotometer [31, 32].

### Assay for Mitochondrial Membrane Potential (MMP, ΔΨm)

Treated HaCaT cells with a dual emission potential-sensitive JC-1 probe (5,5’,6,6’- tetrachloro-1,1’,3,3’-tetraethyl-benzimidazolyl-carbocyanine iodide) in 96-well plates for 30 min at 37°C[33]. And then, determined by a dual-wavelength/double-beam recording spectrophotometer (Ex490nm/Em530, Ex525nm/Em590, Flex Station384, Molecular Devices, USA).

### Detection of ROS

The content of cellular ROS was determined by 2’,7’-dichlorofluorescin diacetate (DCFH2-DA) [34]. In short, HaCaT cells were incubated with 2μM DCFH2-DA for 45 min at 37°C. Next, washed with PBS buffer four times. Finally, the fluorescence intensity was measured by using spectrophotometer (Flex Station 384, Molecular Devices, USA) 485 nm/535 nm [35, 36].

### Determination of ATP content

After various treatments, HaCaT cells were solubilized by Triton X-100(0.5%) with Glycine buffer (100 mM pH 7.4). Intracellular ATP levels were measured by an ATP bio-luminescence assay kit (Sigma) [37]. ATP levels are expressed as micromoles per cell.

### Activity of mitochondrial complexes I, II

HaCaT cells were grown in 100 mm plates. The HaCaT cells were collected by centrifugation at 1500 g for 10 min at 0°C. The collecting pellet was resuspended in mitochondrial isolation buffer (215mM mannitol, 75mM sucrose, 0.1% BSA, 1 mM EGTA, 20mM HEPES, pH 7.2), and then homogenized on ice by a glass homogenizer. The supernatant fraction was centrifuged at 13000 g for 10 min to pellet the mitochondria. Briefly, complex I activity was measured by following the change in NADH for 10min at 340 nm. Next, complex II was determined by using mitochondria (final concentration:30 mg/ml), and then the reaction was started with 10 mM succinate and scanned at 600 nm for 2 min.

## Results

### Preparation and structural characterization of AP

**Fig 1:**
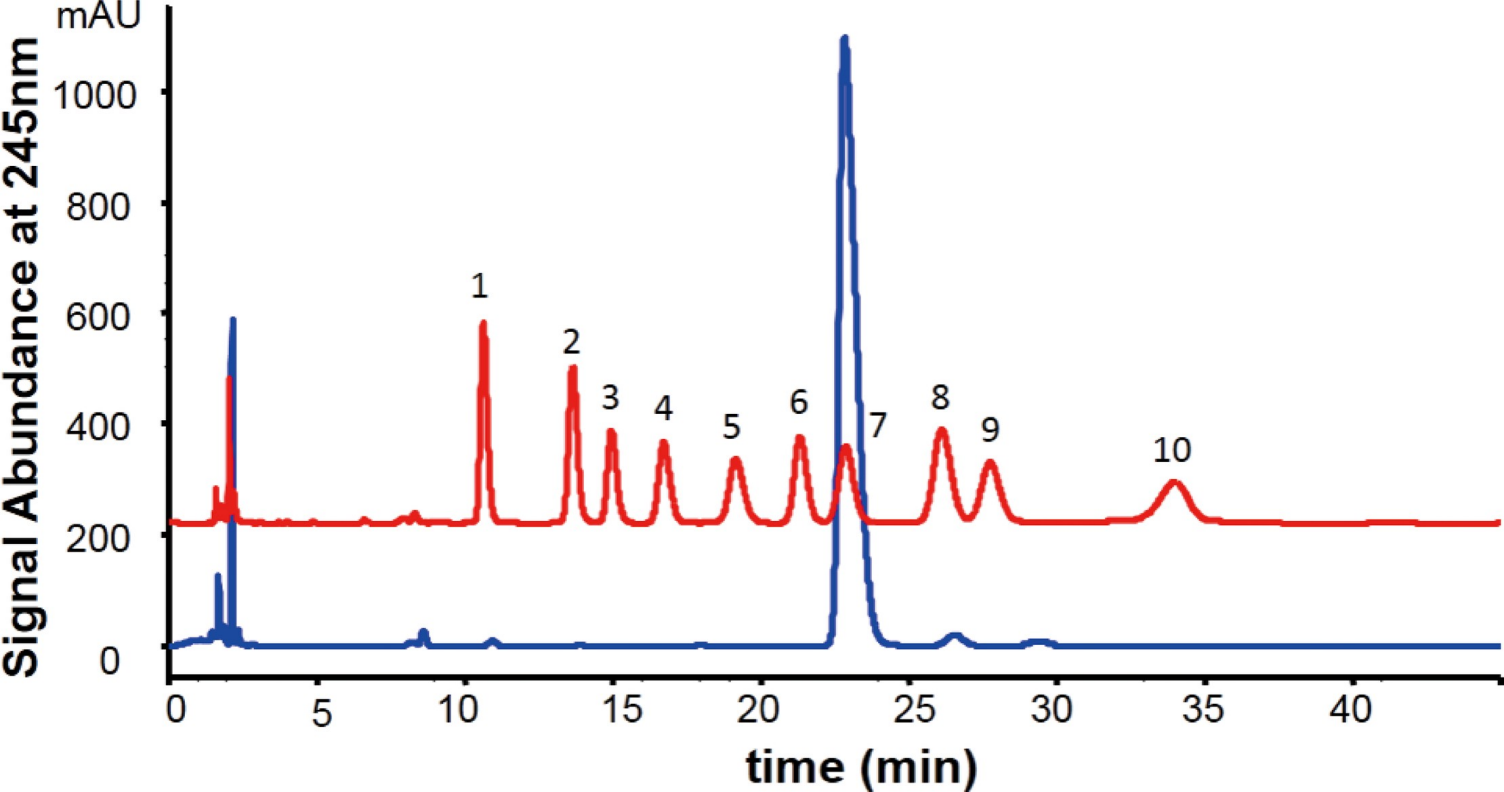
The monosaccharide composition analysis of AP. (1. Man, 2. GlcN, 3. Rha, 4. GlcA, 5. GalA, 6. GalN, 7. Glc, 8. Gal, 9. Xyl, 10. Fuc).

The crude AP was prepared from the dried roots of *Astragalus* membranaceus by using hot water extraction. The crude AP was subsequently separated by Q-Sepharose Fast Flow (QFF) column at 0 M of NaCl to obtain the purified AP. The structural properties of AP including sugar and protein contents, molecular weights (*M*_w_), and monosaccharide compositions were fully characterized. As depicted in Table 1, the total sugar and protein contents of AP were calculated to be 64.9% and 20.1% respectively. The *M*_*w*_ of AP was determined to be 32 KD by HPGPC-MALLS, and the symmetric peak confirmed the purity of the polysaccharide. As shown in Fig 1, PMP-HPLC method was employed to obtain the monosaccharide compositions of AP, and its main component was identified to be glucose with small amounts of mannose and galactose in the ratio of 98.33: 0.33: 1.34 (Glc: Man: Gal).

**Table 1:** General structural characterization of AP.

Sample	Total sugar content	Protein content	*M*_w_	*M*_*w*_/*M*_n_	Monosaccharide molar ratio (%)		
					Glc	Man	Gal
AP	64.9%	20.1%	32kD	1.74	98.33	0.33	1.34

**Fig 2:**
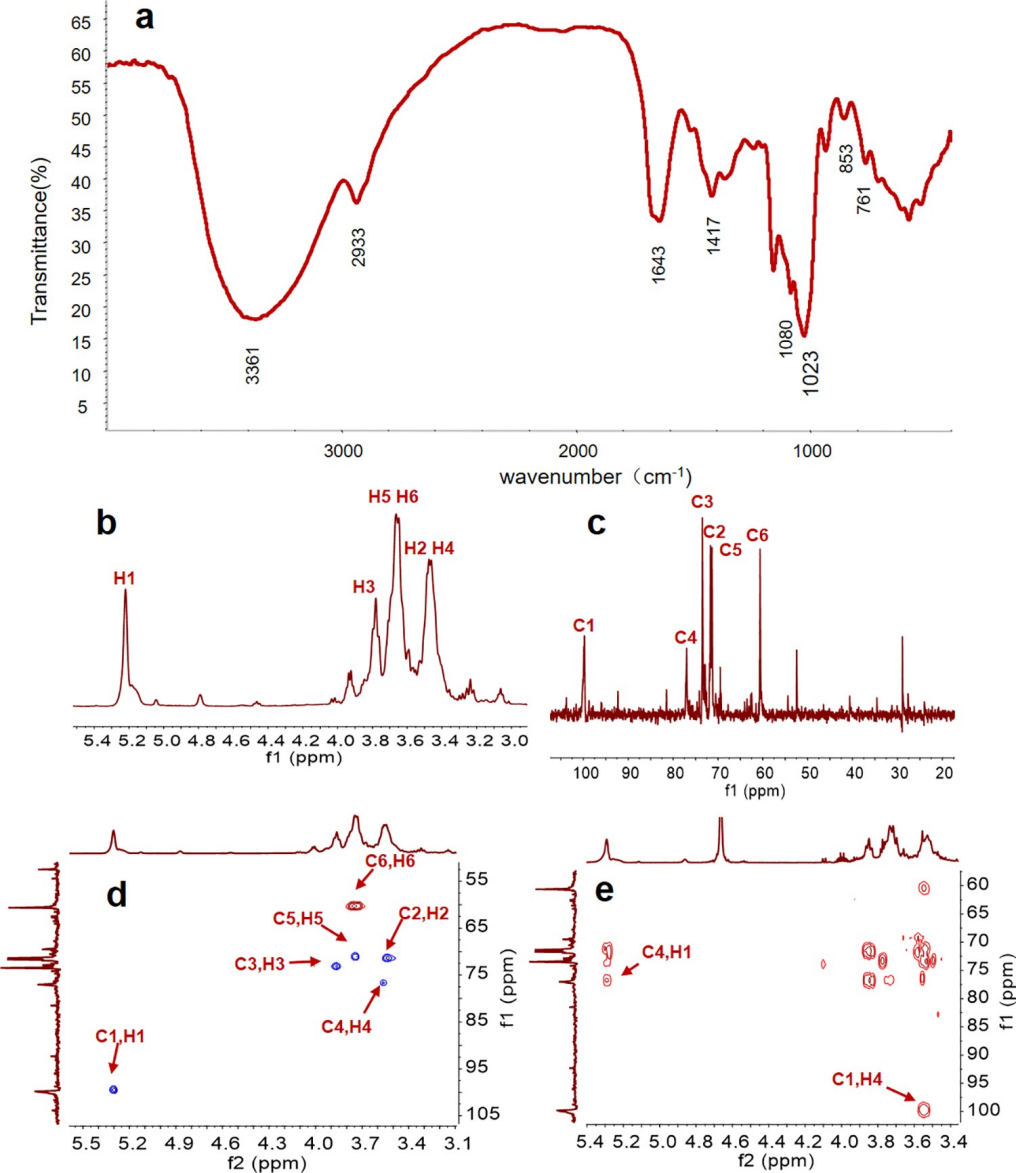
The FT-IR and NMR analysis. (a) FT-IR spectrum of AP. (b) ^1^H-NMR spectrum of AP. (c) ^13^C-NMR spectrum of AP. (d) ^1^H-^13^C HSQC spectrum of AP. (e) ^1^H-^13^C HMBC spectrum of AP.

The FT-IR spectrum of AP is shown in Fig 2(A), the bands at around 3367 cm^−1^ were assigned to symmetric stretching vibration of the hydroxyl groups. The band at 2933 cm^−1^ was attributed to C–H stretching vibration, while the band at around 1417 cm^−1^ was attributed to C–H bending vibration. The polysaccharide hydration vibration absorption peak appeared at 1643 cm^−1^. Meanwhile, the characteristic absorption bands of polysaccharide were found at around 1080 cm^−1^ for C–O–C stretching and 1023 cm^−1^ for C–O stretching. The band at around 853 cm^−1^ was the characteristic absorption of glycosidic bond. The band at around 761 cm^−1^ was stretching vibration of glucose pyranose rings. The structure of AP was further characterized by NMR spectroscopy, and the chemical shifts of protons and carbons were well assigned accordingly. As shown in Fig 2(B), the apparent single peak at 5.22 ppm and minor double peak at 4.91–4.94 ppm corresponded to the H1α and H1β at the reducing end. It is obviously observed that the AP is mainly composed of α configuration. The peak at around 99.80 ppm corresponded to the C1 signals in the ^13^C NMR spectra (Fig 2(C)) of AP. The peaks at 71.69, 73.47, 77.01, 71.35 and 60.63 ppm were assigned to C-2, C-3, C-4, C-5 and C-6, according to ^1^H-^13^C HSQC spectrum (Fig 2(D)). In addition, hydrocarbon remote correlations between C4 and H1 on α-glucan were also clearly identified in ^1^H-^13^C HMBC spectrum (Fig 2(E)). In summary, the AP was preliminarily identified to be an α-(1→4)-glucan after fully structural characterization.

### Cell toxicities of AP

**Fig 3:**
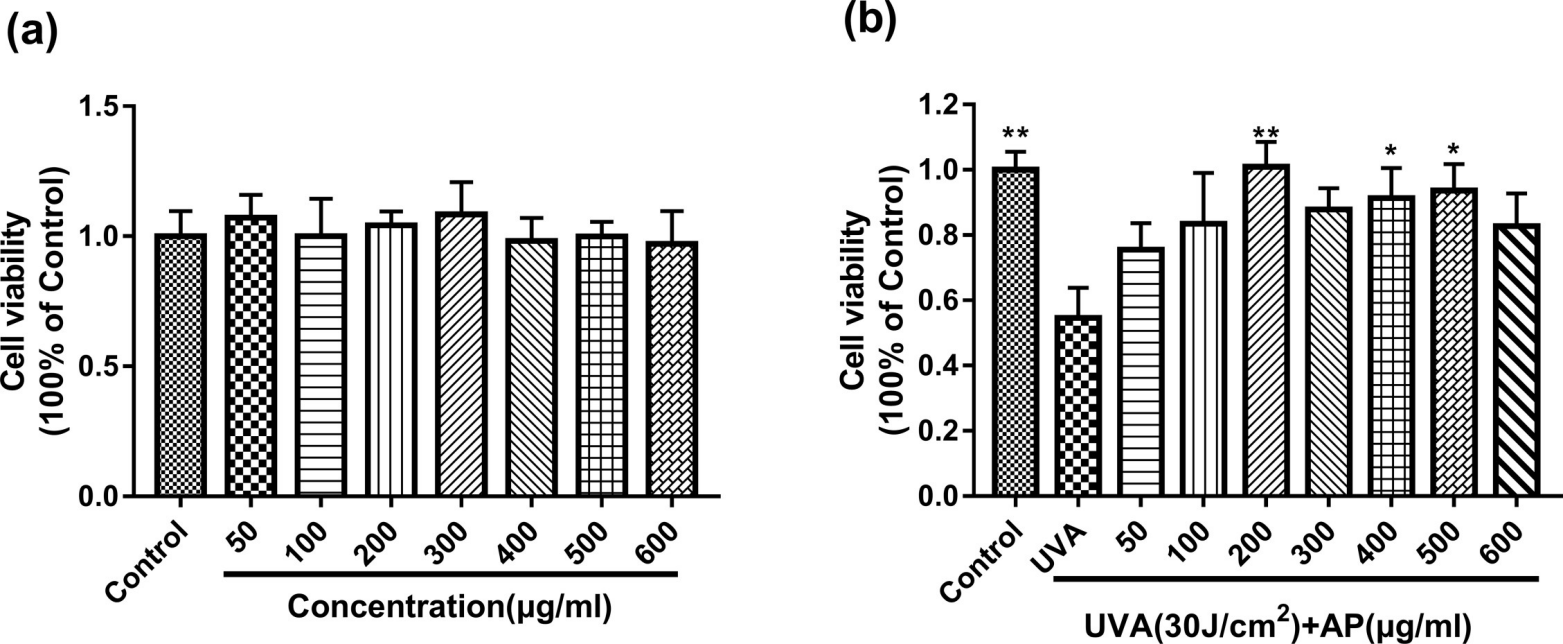
Cytotoxicity of AP on HaCaT cells. (a) Cells were incubated with different concentrations of AP for 48 hours. (b)The cells were pretreated with 0–600μg/ml AP for 48h, and then irradiated with UVA(30J/cm^2^).Cell viability was assessed by MTT assay as described in Materials and Methods. Values are the mean of nine replicates ± S.E.M. *P <0.05 and **P < 0.01 mean significant difference compared to the UVA group.

As shown in Fig 3, the cell toxicities of AP on HaCaT cells was detected by MTT assay. It was demonstrated that AP has no toxic effects on HaCaT cells at concentrations ranging from 50–600μg/ml (Fig 3(A)). HaCaT cells were exposed to different doses of UVA as indicated. Meanwhile, as shown in Fig 3(B), pretreatment of HaCaT cells with 200μg/ml, 400μg/ml, 500μg/ml significantly increased the cell viability. Furthermore, there was no significant increase in the MTT assay of UVA-induced HaCaT cells at a concentration of 600μg/ml compared to 200μg/ml. Therefore, we chose 50–200μg/ml to continue our ΔΨm detection and changes in intracellular ROS content.

### Effect of AP on Mitochondrial Membrane Potential (MMP)

**Fig 4:**
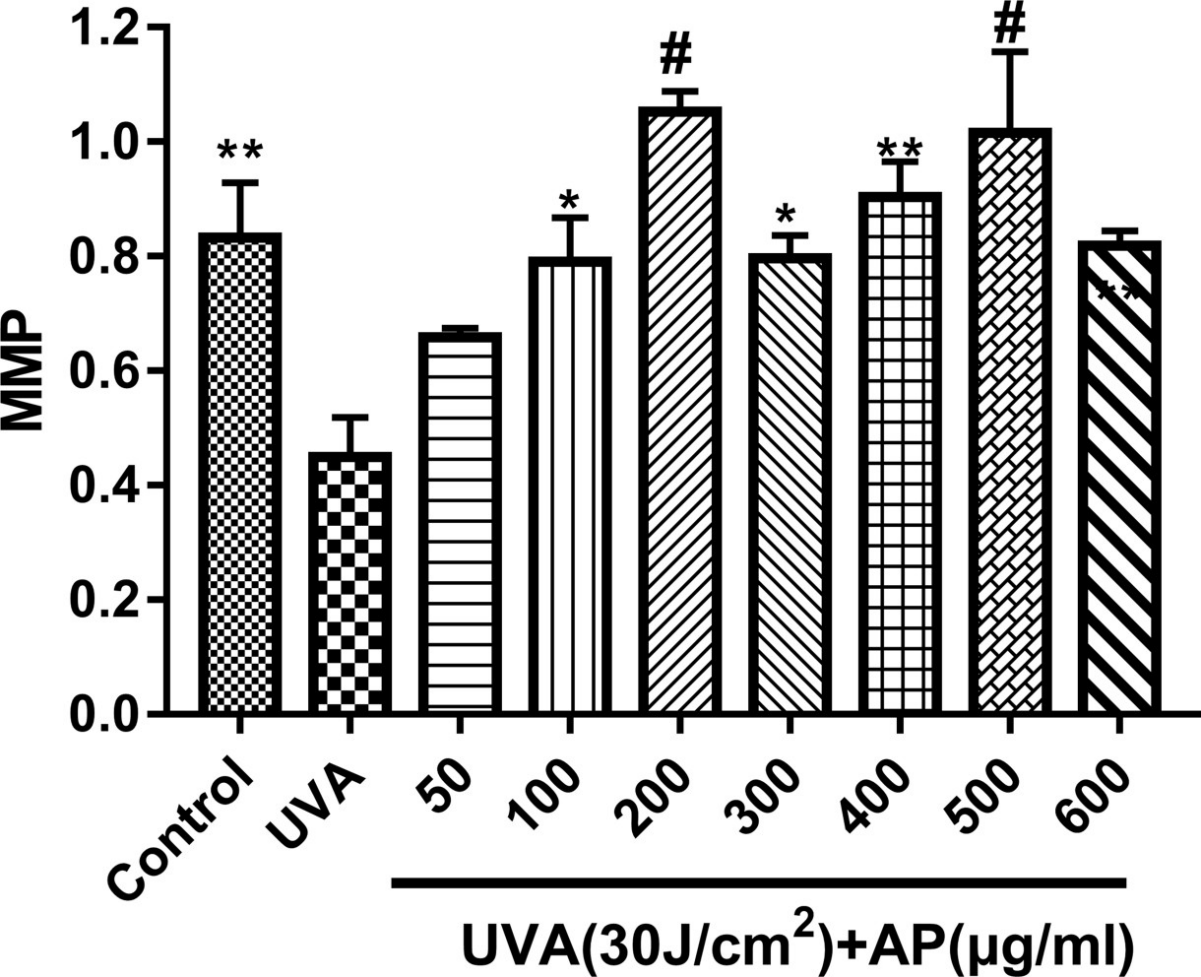
Effect of AP on ΔΨm in UVA-induced HaCaT cells. HaCaT cells were incubated with the indicated concentrations of AP for 48 hours and then irradiated with UVA (30J/cm^2^). After 12 hours, the cells were washed and incubated with JC-1 for ΔΨm assay. *P <0.05, **P < 0.01 and ^#^*P* < 0.0001 mean significant difference from UVA group. Error bars are expressed as mean ± S.E.M. (*n* = 9).

We performed a JC-1 assay to determine changes in mitochondrial membrane potential induced by different doses of UVA. As shown in Fig 4, pretreatment of HaCaT cells with different concentrations of AP significantly inhibited the decrease of ΔΨm in UVA-induced HaCaT cells as determined by JC-1 assay. Within the range of 50–600μg/ml, AP could increase the mitochondrial membrane potential, especially at the concentrations of 100–600μg/ml. In addition, at the concentration of 200μg/ml and 500μg/ml of AP are preferred.

### Effect of AP on ROS production and ATP content

**Fig 5:**
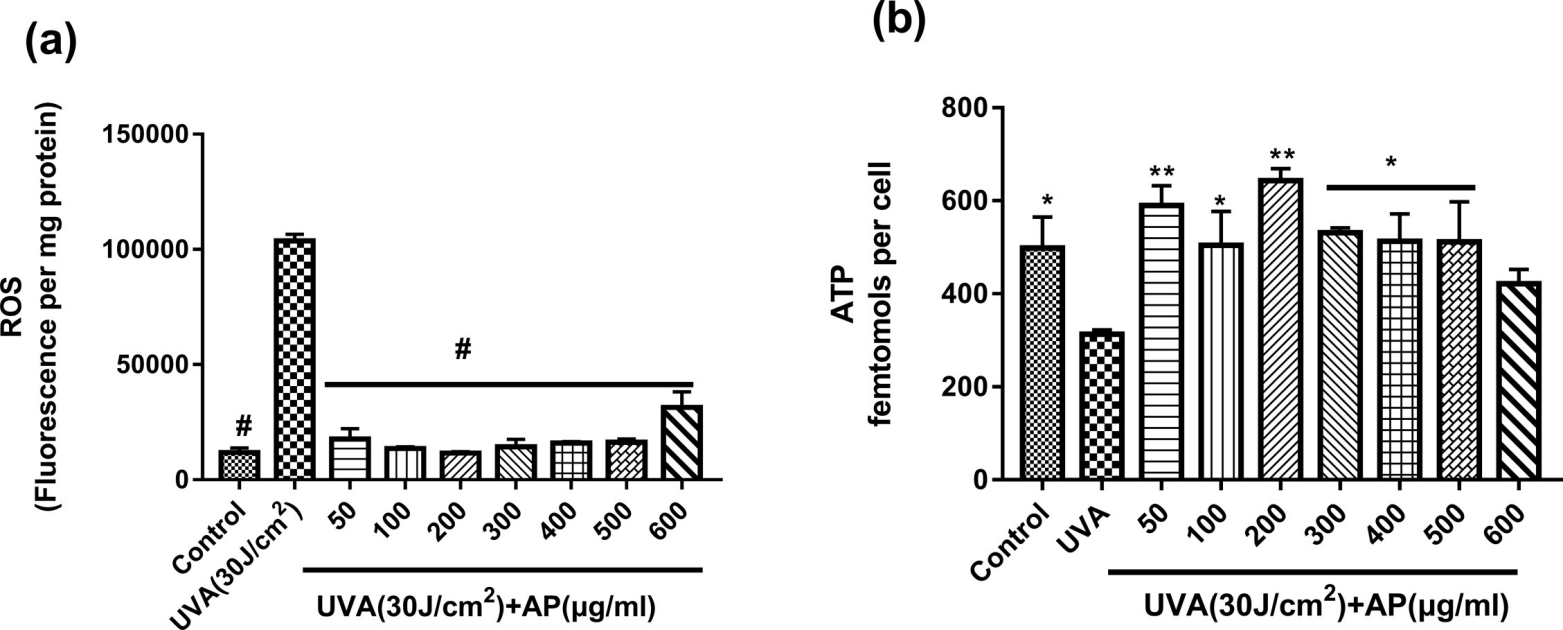
Effect of AP on ROS production and ATP content in UVA-induced HaCaT cells. Cells were incubated with the indicated concentrations of AP for 48 hours and then irradiated with UVA (30 J/cm^2^). After 12 hours, the cells were washed and incubated with CFH2-DA for ROS production assay (a); or with luciferase-based luminescence assay kit for ATP content measurement (b). Values are the mean ± S.E.M of data from at least nine independent experiments. *P <0.05, **P <0.01 and ^#^*P* < 0.0001 compared to the UVA group.

Then ROS production was determined and the results are shown in Fig 5(A). UVA exposure of 30 J/cm^2^ resulted in significant increase in ROS production in HaCaT cells, and pre-treatment with AP significantly inhibited ROS production.

As shown in Figs 3 and 4, the optimal concentration of AP to protect HaCaT cells is 200μg/ml or 500μg/ml. Fig 5(A) shows that the addition of 200μg/ml significantly reduced ROS levels. Fig 5(B) shows that UVA (30J/cm^2^) significantly reduced ATP levels, and 200μg/ml AP pretreatment significantly prevented the decrease in ATP levels in UVA-stimulated HaCaT cells. From Figs 3–5, we could see that 200μg/ml of AP was the most effective concentration for protecting cells from UVA-induced photo-damage in HaCaT cells.

### Effect of AP on mitochondrial functions

**Fig 6:**
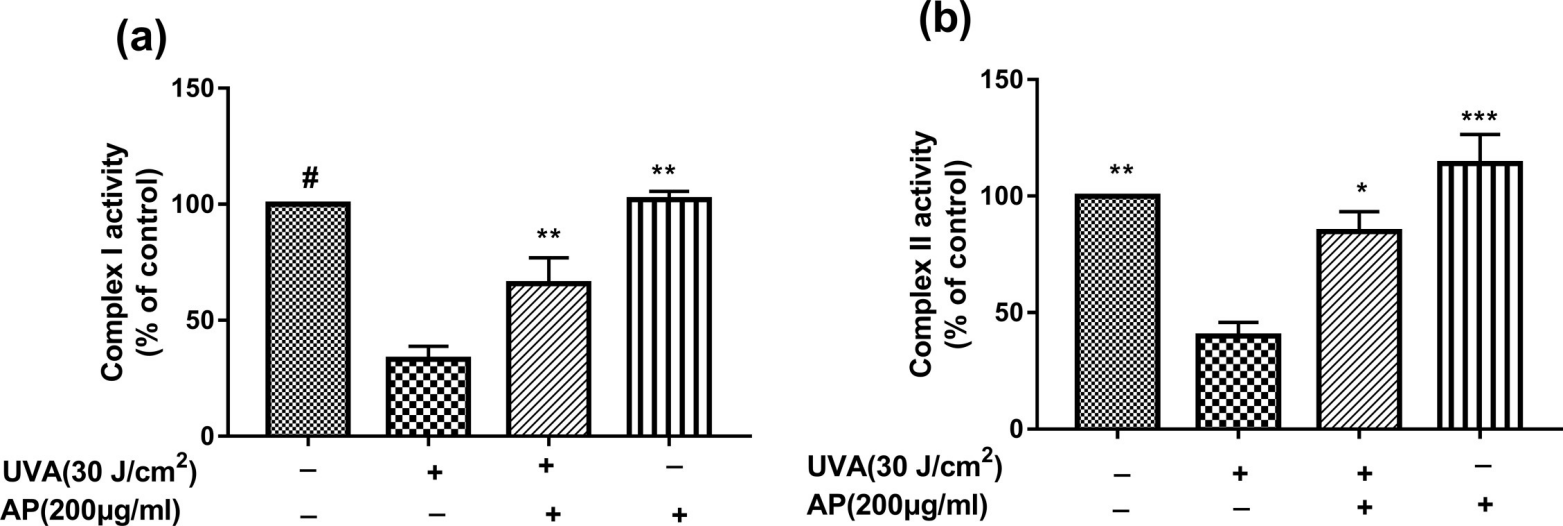
AP could protect HaCaT cells against UVA-induced mitochondrial dysfunction. HaCaT cells were seeded in 6-well plates and incubated with different concentrations of AP for 24 hours. And then, cells were treated with UVA (30J/cm^2^). After 12 hours, the activity of mitochondrial complexes I, II were measured. (a) Determination of mitochondrial complex I activity. (b) Determination of mitochondrial complex II activity. Values are the means ± SEM of the results at least six independent experiments. *P <0.05, **P <0.01, ***P < 0.001 and ^#^*P* < 0.0001 mean significant difference compared to the UVA group.

We next measured whether the increase in ΔΨm and ATP content by AP treatment were associated with enhanced mitochondrial function. It’s showed that AP pretreatment could significantly protect HaCaT cells against UVA-induced reduction in mitochondrial complex I and complex II activities in (Fig 6). From the data showed in Figs 5 and 6, it’s indicated that AP could significantly enhance mitochondrial function, which is not only related to ΔΨm, ATP levels, and complex I-II activity, but also to ROS production.

## Discussion

The skin is the largest organ in the human body. It covers the whole body and is the first barrier to protect the human body from external damage. Skin aging generally has two manifestations: natural aging and photo-damage. Natural aging mainly refers to the programmed aging caused by the irresistible factors in the body. The primary factor of extrinsic skin aging is UV radiation [31]. UVA irradiation can cause significant changes in the stratum corneum, reducing its mechanical integrity and cell cohesion. Furthermore, the UVA radiation destroys the molecular structure of cell lipids and proteins [5]. UV can effect mitochondria and generate ROS to bring about collagen degradation in human skin [38]. Some researches show that excessive ROS actuates NF-κB, which causes the level of cell surface cytokines that promotes photo-damage cytokines such as epidermal growth factor (EGF), interleukin1 (IL1), and tumor necrosis factor alpha. Moreover, these cytokines also contribute to collagen breakdown, DNA strand breaks and apoptosis of cells [39–43]. ROS can destroy DNA strands and trigger death of cells [8, 44]. As show in Figs 5(A) and 3(B), UVA irradiation resulted in a substantial increase in ROS content in HaCaT cells and decrease in cell viability, which was consistent with the former reports [38]. Furthermore, it was found that the treatment of HaCaT cells with AP significantly increased the cell viability and inhibited ROS production as shown in Figs 3(B) and 5(A). Our results indicate that AP can remove excess ROS produced by UVA irradiation to protect HaCaT cells from UVA-induced photo-damage. Moreover, the increased ROS with age can diametrically damage the architectures of the mitochondria itself, such as proteins, lipids, and mtDNA [45–47].

UVA-induced ROS also can generate mtDNA damage, which serves as a marker of skin harm and leads to a decrease in mitochondrial energy metabolism [48]. Moreover, the senescent fibroblasts show a reduction of membrane potential [49]. As shown in Fig 4, different doses of UVA irradiation resulted in a significant decrease in MMP of HaCaT cells. However, Fig 4 shows that treatment of cells with AP significantly reverses this phenomenon. Mitochondria-induced aging diversification may affect mitochondrial respiration. Mitochondria can produce ATP, which is used as chemical energy for most eukaryotic cells. ATP produced by mitochondria also controls cell functions related to differentiation, cell signaling and cell death [50]. Subsequently, Harman established the free radical theory of aging, submitted that the mitochondria’s construction of the superoxide may be middleman of FRTA [51]. This is called the mitochondrial theory of aging. In Fig 5(B), we find that UVA (30J/cm^2^) significantly reduced ATP levels, and 200μg/ml AP pretreatment significantly prevented a decrease in ATP levels of UVA-stimulated HaCaT cells. In the present study, AP increased UVA-induced ATP expression and MMP prevented skin cells from Mitochondrial energy metabolism disorder.

Besides above, complexe I, II, and III are considered to be the main sites attacked by unreasonable ROS [52–55]. Electron transfer through complexes I–IV is administered by the complexes and electron carriers such as Cytochromes C and coenzyme Q10 (CoQ10). The spread of electrons and the ATP synthesis by OxPHOS is successive within mitochondria [56]. However, the defaced mitochondria decrease in function, which in turn produces more ROS. This is known as the ‘‘vicious cycle” which is believed to produce advancing elevated levels of stress [38, 56]. The results of this study indicated that AP pretreatment could significantly protect HaCaT cells against UVA-induced reduction in mitochondrial complex I and complex II activities as shown in Fig 6. Targeting mitochondria can alleviate oxidative stress caused by UVA irradiation. Polyphenols are the most common natural products that have been observed to improve the function of stressed mitochondria, such as resveratrol which has been shown to directly impact mitochondria by modulating the oxidative phosphorylation system [57]. Moreover, the flavonoid is a polyhydroxy phenol that has photo-damage mitigating effects which are associated with preserved mitochondrial function [58, 59]. The same phenomenon has also been observed in Fig 5(A) shows that the anti-oxidative activity of AP may help in protecting the skin from UVA-induced ROS over-expression, which acts as a free radical scavenger. A previous reports clearly showed that the main reasons that influence AgNP-induced Cx43 upregulation include: changes in reactive oxygen content and activation of extracellular signal-regulated kinase and c-Jun N-terminal kinas [60]. Interestingly, we found that the AP protection mechanism is different from silver nanoparticles. In our present study, we found that AP could improve mitochondrial complex I and complex II activities to fight against the reduction of MMP and ATP, which indicates that AP could protect HaCaT cells from photo-damage via targeting to mitochondria.

## Conclusions

In conclusion, our results showed that AP treatment could effectively reduce UVA-induced ROS expression as well as improve the expression of ATP and MMP. Moreover, AP could assist HaCaT cells to fight against the reduction of ATP and MMP through elevating mitochondrial complex I and complex II activities, which indicates that AP may have the protection of skin from photo-damage by targeting to mitochondria.

## Abbreviations

MMP: mitochondrial membrane potential
JC-1: J-Aggregate forming lipophilic cation 55′,6,6′-tetrachloro-1,1′,3,3′ tetraethylbenzimidazolcarbocyanine iodide
BSA: bovine serum albumin
ddH2O: double-distilled water
AP: Astragalus membranaceus polysaccharide
DCFH2-DA: 2’,7’-dichlorofluorescin diacetate

## References

[1] Ruenger TM. Photodermatology, Photoimmunology & Photomedicine. WILEY-BLACKWELL 111 RIVER ST, HOBOKEN 07030–5774, NJ USA; 2014.

[2] Tyrrell R. Solar ultraviolet A radiation: an oxidizing skin carcinogen that activates heme oxygenase-1. Antioxidants & redox signaling. 2004;6(5):835–40. 15345143 10.1089/ars.2004.6.835

[3] Chiang H, Chan S, Chu Y, Wen K. Fisetin Ameliorated Photodamage by Suppressing the Mitogen-Activated Protein Kinase/Matrix Metalloproteinase Pathway and Nuclear Factor-κB Pathways. Journal of Agricultural and Food Chemistry. 2015;63(18):4551–60. 10.1021/jf502500t 25882230

[4] Tyrrell RM. Modulation of gene expression by the oxidative stress generated in human skin cells by UVA radiation and the restoration of redox homeostasis. Photochemical & Photobiological Sciences. 2012;11(1):135–47. 22008784 10.1039/c1pp05222e

[5] Kammeyer A, Luiten R. Oxidation events and skin aging. Ageing research reviews. 2015;21:16–29. 10.1016/j.arr.2015.01.001 25653189

[6] He D, Sun J, Zhu X, Nian S, Liu J. Compound K increases type I procollagen level and decreases matrix metalloproteinase-1 activity and level in ultraviolet-A-irradiated fibroblasts. Journal of the Formosan Medical Association. 2011;110(3):153–60. 10.1016/S0929-6646(11)60025-9 21497278

[7] Lamore SD, Wondrak GT. UVA causes dual inactivation of cathepsin B and L underlying lysosomal dysfunction in human dermal fibroblasts. Journal of Photochemistry and Photobiology B: Biology. 2013;123:1–12. 10.1016/j.jphotobiol.2013.03.007 PMC3710731 23603447

[8] Pustišek N, Šitum M. UV-radiation, apoptosis and skin. Collegium antropologicum. 2011;35(2):339–41. 22220467

[9] Hoeijmakers JH. DNA damage, aging, and cancer. New England Journal of Medicine. 2009;361(15):1475–85. 10.1056/NEJMra0804615 19812404

[10] Quiroga RM. Anti-aging medicine as it relates to dermatology. Cosmetic Dermatology: Springer; 2005 p. 1–16.

[11] Ganceviciene R, Liakou AI, Theodoridis A, Makrantonaki E, Zouboulis CC. Skin anti-aging strategies. Dermato-endocrinology. 2012;4(3):308–19. 10.4161/derm.22804 23467476 PMC3583892

[12] Wallace DC. A mitochondrial paradigm of metabolic and degenerative diseases, aging, and cancer: a dawn for evolutionary medicine. Annu Rev Genet. 2005;39:359–407. 10.1146/annurev.genet.39.110304.095751 16285865 PMC2821041

[13] Clayton DA, Doda JN, Friedberg EC. The absence of a pyrimidine dimer repair mechanism in mammalian mitochondria. Proceedings of the National Academy of Sciences. 1974;71(7):2777–81. 10.1073/pnas.71.7.2777 PMC388554 4212385

[14] Harman D. Free radical theory of aging: the “free radical” diseases. Age. 1984;7(4):111–31.

[15] Harbottle A, Birch-Machin M. Real-time PCR analysis of a 3895 bp mitochondrial DNA deletion in nonmelanoma skin cancer and its use as a quantitative marker for sunlight exposure in human skin. British journal of cancer. 2006;94(12):1887 10.1038/sj.bjc.6603178 16721366 PMC2361359

[16] Naidoo K, Hanna R, Birch‐Machin MA. What is the role of mitochondrial dysfunction in skin photoaging? Experimental dermatology. 2018;27(2):124–8. 10.1111/exd.13476 29197123

[17] Birch‐Machin M, Russell E, Latimer J. Mitochondrial DNA damage as a biomarker for ultraviolet radiation exposure and oxidative stress. British Journal of Dermatology. 2013;169:9–14. 10.1111/bjd.12207 23786615

[18] Kagan VE, Bayır HA, Belikova NA, Kapralov O, Tyurina YY, Tyurin VA, et al Cytochrome c/cardiolipin relations in mitochondria: a kiss of death. Free Radical Biology and Medicine. 2009;46(11):1439–53. 10.1016/j.freeradbiomed.2009.03.004 19285551 PMC2732771

[19] Liu G-Y, Moon SH, Jenkins CM, Li M, Sims HF, Guan S, et al The phospholipase iPLA2γ is a major mediator releasing oxidized aliphatic chains from cardiolipin, integrating mitochondrial bioenergetics and signaling. Journal of Biological Chemistry. 2017;292(25):10672–84. 10.1074/jbc.M117.783068 28442572 PMC5481572

[20] Finkel T, Menazza S, Holmström KM, Parks RJ, Liu J, Sun J, et al The ins and outs of mitochondrial calcium. Circulation research. 2015;116(11):1810–9. 10.1161/CIRCRESAHA.116.305484 25999421 PMC6296495

[21] Park K, Lee J-H. Protective effects of resveratrol on UVB-irradiated HaCaT cells through attenuation of the caspase pathway. Oncology reports. 2008;19(2):413–7. 18202789

[22] Cho S, Lee DH, Won C-H, Kim SM, Lee S, Lee M-J, et al Differential effects of low-dose and high-dose beta-carotene supplementation on the signs of photoaging and type I procollagen gene expression in human skin in vivo. Dermatology. 2010;221(2):160–71. 10.1159/000305548 20516658

[23] Petruk G, Raiola A, Del Giudice R, Barone A, Frusciante L, Rigano MM, et al An ascorbic acid-enriched tomato genotype to fight UVA-induced oxidative stress in normal human keratinocytes. Journal of Photochemistry and Photobiology B: Biology. 2016;163:284–9. 10.1016/j.jphotobiol.2016.08.047 27599115

[24] Kim KH, Park SJ, Lee YJ, Lee JE, Song CH, Choi SH, et al Inhibition of UVB‐Induced Skin Damage by Exopolymers from Aureobasidium pullulans SM‐2001 in Hairless Mice. Basic & clinical pharmacology & toxicology. 2015;116(2):73–86. 24964914 10.1111/bcpt.12288

[25] Röding J. Beta-(1, 3)-Beta-(1, 4)-Glucan as Carrier for Chemical Substances. Google Patents; 2007.

[26] Griesbach U, Wachter R, Ansmann A, Fabry B, Engstad RE. Use of water-soluble β-(1, 3) glucans as agents for producing therapeutic skin treatment agents. Google Patents; 2005.

[27] Del Giudice R, Petruk G, Raiola A, Barone A, Monti DM, Rigano MM. Carotenoids in fresh and processed tomato (Solanum lycopersicum) fruits protect cells from oxidative stress injury. Journal of the Science of Food and Agriculture. 2017;97(5):1616–23. 10.1002/jsfa.7910 27434883

[28] Bian Y-Y, Guan J, Bi Z-M. Studies on chemical constituents of Astragalus membranaceus (Fisch.) Bge. var. mongholicus (Bge.) Hsiao. CHINESE PHARMACEUTICAL JOURNAL-BEIJING-. 2006;41(16):1217.

[29] Jiangwei M, Zengyong Q, Xia X. Aqueous extract of Astragalus mongholicus ameliorates high cholesterol diet induced oxidative injury in experimental rats models. Journal of Medicinal Plants Research. 2011;5(5):855–8.

[30] Chen Y, Liu X, Wu L, Tong A, Zhao L, Liu B, et al Physicochemical characterization of polysaccharides from Chlorella pyrenoidosa and its anti-ageing effects in Drosophila melanogaster. Carbohydrate polymers. 2018;185:120–6. 10.1016/j.carbpol.2017.12.077 29421048

[31] Zhang R, Chen J, Zhang X. Extraction of intracellular protein from Chlorella pyrenoidosa using a combination of ethanol soaking, enzyme digest, ultrasonication and homogenization techniques. Bioresource technology. 2018;247:267–72. 10.1016/j.biortech.2017.09.087 28950135

[32] Chiang H-M, Chen C-W, Lin T-Y, Kuo Y-H. N-phenethyl caffeamide and photodamage: Protecting skin by inhibiting type I procollagen degradation and stimulating collagen synthesis. Food and chemical toxicology. 2014;72:154–61. 10.1016/j.fct.2014.07.007 25019243

[33] Wu P-Y, Huang C-C, Chu Y, Huang Y-H, Lin P, Liu Y-H, et al Alleviation of ultraviolet B-induced photodamage by Coffea arabica extract in human skin fibroblasts and hairless mouse skin. International journal of molecular sciences. 2017;18(4):782. 10.3390/ijms18040782 PMC5412366 28387707

[34] Tirosh O, Sen C, Roy S, Packer L. Cellular and mitochondrial changes in glutamate-induced HT4 neuronal cell death. Neuroscience. 2000;97(3):531–41. 10.1016/s0306-4522(00)00028-2 10828535

[35] Cathcart R, Schwiers E, Ames BN. Detection of picomole levels of hydroperoxides using a fluorescent dichlorofluorescein assay. Analytical biochemistry. 1983;134(1):111–6. 10.1016/0003-2697(83)90270-1 6660480

[36] Wölfle U, Esser PR, Simon-Haarhaus B, Martin SF, Lademann J, Schempp CM. UVB-induced DNA damage, generation of reactive oxygen species, and inflammation are effectively attenuated by the flavonoid luteolin in vitro and in vivo. Free Radical Biology and Medicine. 2011;50(9):1081–93. 10.1016/j.freeradbiomed.2011.01.027 21281711

[37] Patanè G, Anello M, Piro S, Vigneri R, Purrello F, Rabuazzo AM. Role of ATP production and uncoupling protein-2 in the insulin secretory defect induced by chronic exposure to high glucose or free fatty acids and effects of peroxisome proliferator-activated receptor-γ inhibition. Diabetes. 2002;51(9):2749–56. 10.2337/diabetes.51.9.2749 12196468

[38] Chu Y, Wu P-Y, Chen C-W, Lyu J-L, Liu Y-J, Wen K-C, et al Protective Effects and Mechanisms of N-Phenethyl Caffeamide from UVA-Induced Skin Damage in Human Epidermal Keratinocytes through Nrf2/HO-1 Regulation. International journal of molecular sciences. 2019;20(1):164. 10.3390/ijms20010164 PMC6337442 30621167

[39] Matsumura Y, Ananthaswamy HN. Toxic effects of ultraviolet radiation on the skin. Toxicology and applied pharmacology. 2004;195(3):298–308. 10.1016/j.taap.2003.08.019 15020192

[40] Chiang H-M, Chan S-Y, Chu Y, Wen K-C. Fisetin ameliorated photodamage by suppressing the mitogen-activated protein kinase/matrix metalloproteinase pathway and nuclear factor-κB pathways. Journal of agricultural and food chemistry. 2015;63(18):4551–60. 10.1021/jf502500t 25882230

[41] Fisher DE, James WD. Indoor tanning—science, behavior, and policy. New England Journal of Medicine. 2010;363(10):901–3. 10.1056/NEJMp1005999 20818900 PMC3951814

[42] Trocoli A, Djavaheri-Mergny M. The complex interplay between autophagy and NF-κB signaling pathways in cancer cells. American journal of cancer research. 2011;1(5):629 21994903 PMC3189824

[43] Ruland J, Mak TW. Transducing signals from antigen receptors to nuclear factor κB. Immunological reviews. 2003;193(1):93–100. 12752674 10.1034/j.1600-065x.2003.00049.x

[44] Halliwell B, Gutteridge J. Free radicals in biology and medicine: Oxford University Press. Inc, New York 1999.

[45] Chen JJ, Yu BP. Alterations in mitochondrial membrane fluidity by lipid peroxidation products. Free Radical Biology and Medicine. 1994;17(5):411–8. 10.1016/0891-5849(94)90167-8 7835747

[46] Sohal RS, Dubey A. Mitochondrial oxidative damage, hydrogen peroxide release, and aging. Free Radical Biology and Medicine. 1994;16(5):621–6. 10.1016/0891-5849(94)90062-0 8026805

[47] Agarwal S, Sohal RS. DNA oxidative damage and life expectancy in houseflies. Proceedings of the National Academy of Sciences. 1994;91(25):12332–5. 10.1073/pnas.91.25.12332 PMC45431 7991627

[48] Berneburg M, Gremmel T, Kürten V, Schroeder P, Hertel I, Von Mikecz A, et al Creatine supplementation normalizes mutagenesis of mitochondrial DNA as well as functional consequences. Journal of General Internal Medicine. 2005;20(5):213–20. 16098029 10.1111/j.0022-202X.2005.23806.x

[49] Mammone T, Gan D, Foyouzi‐Youssefi R. Apoptotic cell death increases with senescence in normal human dermal fibroblast cultures. Cell biology international. 2006;30(11):903–9. 10.1016/j.cellbi.2006.06.010 16904918

[50] Zwerschke W, Mazurek S, Stöckl P, Hütter E, Eigenbrodt E, Jansen-Dürr P. Metabolic analysis of senescent human fibroblasts reveals a role for AMP in cellular senescence. Biochemical Journal. 2003;376(Pt 2):403 10.1042/BJ20030816 12943534 PMC1223775

[51] Harman D. The biologic clock: the mitochondria? Journal of the American Geriatrics Society. 1972;20(4):145–7. 10.1111/j.1532-5415.1972.tb00787.x 5016631

[52] Chen Q, Vazquez EJ, Moghaddas S, Hoppel CL, Lesnefsky EJ. Production of reactive oxygen species by mitochondria central role of complex III. Journal of Biological Chemistry. 2003;278(38):36027–31. 10.1074/jbc.M304854200 12840017

[53] Turrens JF. Superoxide production by the mitochondrial respiratory chain. Bioscience reports. 1997;17(1):3–8. 10.1023/a:1027374931887 9171915

[54] Turrens JF. Mitochondrial formation of reactive oxygen species. The Journal of physiology. 2003;552(2):335–44. 14561818 10.1113/jphysiol.2003.049478 PMC2343396

[55] Barja G. Mitochondrial oxygen radical generation and leak: sites of production in states 4 and 3, organ specificity, and relation to aging and longevity. Journal of bioenergetics and biomembranes. 1999;31(4):347–66. 10.1023/a:1005427919188 10665525

[56] Linnane A, Ozawa T, Marzuki S, Tanaka M. Mitochondrial DNA mutations as an important contributor to ageing and degenerative diseases. The Lancet. 1989;333(8639):642–5. 10.1016/s0140-6736(89)92145-4 2564461

[57] de Oliveira MR, Nabavi SF, Manayi A, Daglia M, Hajheydari Z, Nabavi SM. Resveratrol and the mitochondria: From triggering the intrinsic apoptotic pathway to inducing mitochondrial biogenesis, a mechanistic view. Biochimica et Biophysica Acta (BBA)-General Subjects. 2016;1860(4):727–45. 26802309 10.1016/j.bbagen.2016.01.017

[58] Farris P, Yatskayer M, Chen N, Krol Y, Oresajo C. Evaluation of efficacy and tolerance of a nighttime topical antioxidant containing resveratrol, baicalin, and vitamin e for treatment of mild to moderately photodamaged skin. Journal of drugs in dermatology: JDD. 2014;13(12):1467–72. 25607790

[59] de Oliveira MR, Nabavi SF, Habtemariam S, Orhan IE, Daglia M, Nabavi SM. The effects of baicalein and baicalin on mitochondrial function and dynamics: a review. Pharmacological Research. 2015;100:296–308. 10.1016/j.phrs.2015.08.021 26318266

[60] Qin Y, Han L, Yang D, Wei H, Liu Y, Xu J, et al Silver nanoparticles increase connexin43‐mediated gap junctional intercellular communication in HaCaT cells through activation of reactive oxygen species and mitogen‐activated protein kinase signal pathway. Journal of Applied Toxicology. 2018;38(4):564–74. 10.1002/jat.3563 29235124

