# Supplementary material for: Photoprotective effect of Astragalus membranaceus polysaccharide on UVA-induced damage in HaCaT cells
Source: PLoS One. 2020 Jul 21;15(7):e0235515. doi: 10.1371/journal.pone.0235515 (PMC7373302; doi:10.1371/journal.pone.0235515)
Supplement: Attachment — Submitted filename: Response to Reviewers .doc [file pone.0235515.s001.doc]

# Response to Editor and Reviewer Comments

Dear Editors:

On behalf of my co-authors, we thank you very much for giving us an opportunity to revise our manuscript. And the positive and constructive comments from editor and reviewers on our manuscript entitled “Protective effects of *Astragalus membranaceus* polysaccharide on UVA-induced photo-aging in HaCaT cells”(ID: PONE-D-20-05967) were highly appreciated These comments are all valuable and very helpful for revising and improving our paper, as well as the important guiding significance to our researches. We have studied comments carefully and have made correction which we hope to meet with approval. Revised portion are marked in red with underline in the paper. We have updated Funding Statement and Competing Interests Statementaccording to all PLOS ONE policies. The main corrections in the paper and the responds to the reviewer’s comments are listed as follows. We would like to express our great appreciation to you and reviewers for comments on our paper again. And we are Looking forward to hearing from you.
Yours sincerely,
Jiejie Hao
E-mail: 2009haojie@ouc.edu.cn

Additional Editor Comments :
• Give the structure of the isolated compound.
• The reported photoprotection is due to scavenging UVA-induced ROS or the sunscreen property of the compound?. The authors may have to report the SPF value of the isolated compound.
• How do the authors irradiated the HaCaT cells? How many cells used for irradiation. Give the brief methodology of the UVA treatment and/or the polysaccharide treatment.
• The authors have not analyzed UVA-mediated photoaging specific markers like matrix metalloproteinases expression or collagen degradation in this study. Therefore, the title may not be like “UVA-induced photoaging”. Either include effect of AP on certain photoaging markers or re-write the title as “Photoprotective effect of AP….”
• Additional experiments on the effect of AP against UVA-induced mitDNA damage may further improve the novelty of the present work.

**Response:** Special thanks for your comments. Your suggestions are very valuable and helpful to our further study.

1.We are greatly appreciated your professional comments. we provide the structure of the isolated compound.

Fig S1. The structure of AP

2.Great question! It’s valuable to investigate the intrinsic photoprotection mechanisms of AP we observed in our experiments. So we have planned to compare the protective effects between the present AP and enzyme-degraded AP oligosaccharides, and designed to probe both of them by FITC and observe their process of entering the skin cells. Meanwhile, we also designed to detect the ROS production level along with the time of extra- and inter-cells. We hope these experiments would help us know the question you bring for us. Thanks again.

3. First, we planted 8000 cells per well in a 96-well plate and placed it in a constant temperature cell incubator for 12 hours; then, added different concentrations of AP and placed it in a constant temperature cell incubator for 48 hours. After the incubation, the 96-well plate was placed in a UV cross-linker and irradiated with 30J/cm^2^ UVA; after the irradiation was completed, the medium was replaced and placed in a constant temperature cell incubator for 12 hours, and then, the biochemical indicators were measured.

4. Thanks a lot for your helpful and constructive suggestions. We have re-write the title as “Photoprotective effect of *Astragalus membranaceus* polysaccharide on UVA-induced damage in HaCaT cells”
5. We are greatly appreciated your professional comments. We will detect the UVA-induced mtDNA damage in our further investigation to compare the difference between the polysaccharides of AP and its oligosaccharides.

Reviewer #1: This is an interesting article on the effect of Astragalus membranaceus polysaccharide (AP) on photo-aging of HaCaT cells by reducing UVA-induced intracellular reactive oxygen species (ROS) production and mitochondrial membrane potential, thereby altering ATP content. The authors could also refer to the literature on the positive effect of silver nanoparticles on the reduction of UV-induced damage to HaCaT cells and comment on whether the AP protective mechanism is different from that of silver nanoparticles.

**Response:** Thanks very much for your suggestion. We have made additions marked in red in the revised manuscript according to your suggestion.

Please include both an updated Funding Statement and Competing Interests Statement in your cover letter. We will change the online submission form on your behalf.

**Response:** Special thanks for your comments. We have updated Funding Statement and Competing Interests Statementaccording to all PLOS ONE policies in line 338-344 and line 349-352.

**Funding**

This work was supported by National Natural Science Foundation of China (81973231, 81402982, 31670811), National Science and Technology Major Project for Significant New Drugs Development (2018ZX09735004). The funders had no role in study design, data collection and analysis, decision to publish, or preparation of the manuscript.

**Competing interests**

The authors of Qiong Li, Depeng Wang, Donghui Bai , Chao Cai, Jia Li, Chengxiu Yan, Shuai Zhang, Jiejie Hao and Guangli Yu declare that they have no competing interests; Zhiyun Wu also provides a Competing Interests Statement declaring that she has no competing interests.
